# Surface and Structural Studies of Age-Related Changes in Dental Enamel: An Animal Model

**DOI:** 10.3390/ma15113993

**Published:** 2022-06-03

**Authors:** Izabela Świetlicka, Ewa Tomaszewska, Siemowit Muszyński, Michał Świetlicki, Tomasz Skrzypek, Wojciech Grudziński, Wiesław I. Gruszecki, Daniel Kamiński, Monika Hułas-Stasiak, Marta Arczewska

**Affiliations:** 1 Department of Biophysics, Faculty of Environmental Biology, University of Life Sciences, Akademicka 13, 20-033 Lublin, Poland; siemowit.muszynski@up.lublin.pl; 2 Department of Animal Physiology, Faculty of Veterinary Medicine, University of Life Sciences, Akademicka 12, 20-033 Lublin, Poland; ewarst@interia.pl; 3 Department of Applied Physics, Faculty of Mechanical Engineering, Lublin University of Technology, Nadbystrzycka 36, 20-618 Lublin, Poland; m.swietlicki@pollub.pl; 4 Laboratory of Confocal and Electron Microscopy, Centre for Interdisciplinary Research, Faculty of Science and Health, John Paul II Catholic University of Lublin, Konstantynów 1J, 20-708 Lublin, Poland; tomasz.skrzypek@kul.pl; 5 Department of Biophysics, Institute of Physics, Maria Curie-Sklodowska University, Maria Sklodowska-Curie 1, 20-031 Lublin, Poland; wojciech.grudzinski@mail.umcs.pl (W.G.); wieslaw.gruszecki@mail.umcs.pl (W.I.G.); 6 Department of Crystallography, Faculty of Chemistry, Maria Curie-Sklodowska University, Maria Sklodowska-Curie 2, 20-031 Lublin, Poland; daniel.kaminski@umcs.pl; 7 Department of Functional Anatomy and Cytobiology, Maria Curie-Sklodowska University, Akademicka 19, 20-031 Lublin, Poland; monika.hulas-stasiak@mail.umcs.pl

**Keywords:** enamel, XRD, FTIR, Raman spectroscopy, AFM, modulus of elasticity, EDS, SEM, ANNs

## Abstract

In the animal kingdom, continuously erupting incisors provided an attractive model for studying the enamel matrix and mineral composition of teeth during development. Enamel, the hardest mineral tissue in the vertebrates, is a tissue sensitive to external conditions, reflecting various disturbances in its structure. The developing dental enamel was monitored in a series of incisor samples extending the first four weeks of postnatal life in the spiny mouse. The age-dependent changes in enamel surface morphology in the micrometre and nanometre-scale and a qualitative assessment of its mechanical features were examined by applying scanning electron microscopy (SEM) and atomic force microscopy (AFM). At the same time, structural studies using XRD and vibrational spectroscopy made it possible to assess crystallinity and carbonate content in enamel mineral composition. Finally, a model for predicting the maturation based on chemical composition and structural factors was constructed using artificial neural networks (ANNs). The research presented here can extend the existing knowledge by proposing a pattern of enamel development that could be used as a comparative material in environmental, nutritional, and pharmaceutical research.

## 1. Introduction

Higher vertebrates have evolved a process of biomineralisation resulting in tooth enamel with a unique morphology and a hierarchical nanocomposite structure, reflected in its remarkable physical properties, making it the hardest material in the biological world. Therefore, understanding the mechanisms involved in biomineralisation is of great importance not only from a scientific point of view but can also be a source of inspiration for developing new materials, technologies and therapeutic methods to improve the quality of life. Therefore, a vital issue concerning biomineralisation is to clarify the initiation and the progression of this multi-stage process.

Enamel consists of 95% mineral phase, 4% water and 1% organic matter [1]. The organic phase comprises proteins (amelogenins, ameloblastin, enamelin, and tuftelin) and minor concentrations of proteoglycans and lipids [2,3,4], while the mineral part is built with hydroxyapatite (HA) crystals (Ca_10_(PO_4_)_6_(OH)_2_) [1]. Enamel is formed in the highly ordered process called amelogenesis, which runs through three functional phases: presecretory, secretory, and maturation [2,3]. During the prenatal period, ameloblasts differentiate from the dental epithelial progenitor cells [5], which causes the sequential expression of tissue-specific genes whose products form the enamel extracellular protein matrix. Then, the process of amelogenesis ends with the formation of HA crystals resulting in the reduction of organic content and complete mineralisation of the enamel [6]. During amelogenesis, due to hereditary, systemic or environmental factors [7], some alterations may appear in the structure of the enamel. These changes are manifested, among others, by modification of enamel’s colour and texture, its mechanical properties such as strength and hardness, and crystal or molecular structure [5,6]. Furthermore, due to the rapid degradation of the matrix proteins once formed, enamel crystals cannot be remodelled [8] or repaired [9]. Then, it could be assumed that without the influence of external factors, the enamel remains in the obtained form for the further part of life. The exceptions are rodents whose incisors are constantly growing because ameloblasts retain their functions and ensure the continuous formation of enamel [10].

All modern rodents possess a pair of hypselodont, chisel-like incisors in the upper and lower jaws, with a labial surface covered with a thin enamel layer (about 0.1 mm). Although rodent incisors are morphologically distinct from humans’, enamel and dentin are histologically the same, which helps to understand the development process in human teeth [11,12]. Furthermore, due to the high growth rate, the incisors serve as a model of general tissue growth processes and cellular interactions mechanisms [13]. Apart from that, rodent incisors are examined in the context of environmental pollution and, considering their morphology is related to diet, in nutritional experiments [14,15,16,17].

Among the animal models used in a broad range of research fields, the spiny mouse (*Acomys cahirinus*) should be mentioned. *Acomys* is a small rodent species living throughout Africa, the Middle East and Southwest Asia [18] and is widely used as a model animal in a wide range of experiments. For example, the spiny mouse is currently used for diabetes examination because being fed high-fat diets develop obesity and hyperglycaemia. Additionally, *A. cahirinus* has a relatively long gestation period (approximately 39 days), and offspring are born in an advanced stage of development [19], which makes the species an ideal model for foetal and neonatal studies, organogenesis and late pregnancy biology [20,21,22]. Furthermore, they have recently been shown to have remarkable healing and regeneration capabilities [19,23,24] and to undergo menstruation, which is a rare characteristic in mammals [25].

Nowadays, teeth constitute a classical model for studying signalling pathways and mediating interactions between cells and tissues in organ development, homeostasis and regeneration [26]. In addition, tooth analyses are also increasingly used in the biomedical field to obtain individual histories of health and exposure to adversities during prenatal and postnatal development [27,28]. The application of teeth as a biomarker uses several unique features of tooth formation, such as a known timeline, the absence of turnover, or diffusion of building and nutritional substances from the blood vessels [29], hence the influence of external factors such as dietary, ionising radiation, heavy metals, drugs or the burden of certain diseases. For example, diabetes is clearly visible as it may cause a significant impact on the teeth, especially enamel tissue, by disrupting its functions and causing a number of changes in their structure [30,31,32,33,34].

This study sought to add to current insights by proposing a scheme of enamel development, which could be used as a comparative material in environmental, nutritional and pharmaceutical studies. To accomplish this goal, the enamel of the *Acomys* incisors was examined and characterised for surface features, chemical composition, crystalline arrangement, and molecular structure during the first four weeks of life.

## 2. Materials and Methods

The Ist Local Ethics Committee approved the experiment for Animal Experimentation of Maria Curie-Sklodowska University in Lublin, Poland (reference number 8/2014). The experiment was conducted according to EU Directive 2010/63/EU.

**Figure 1:**
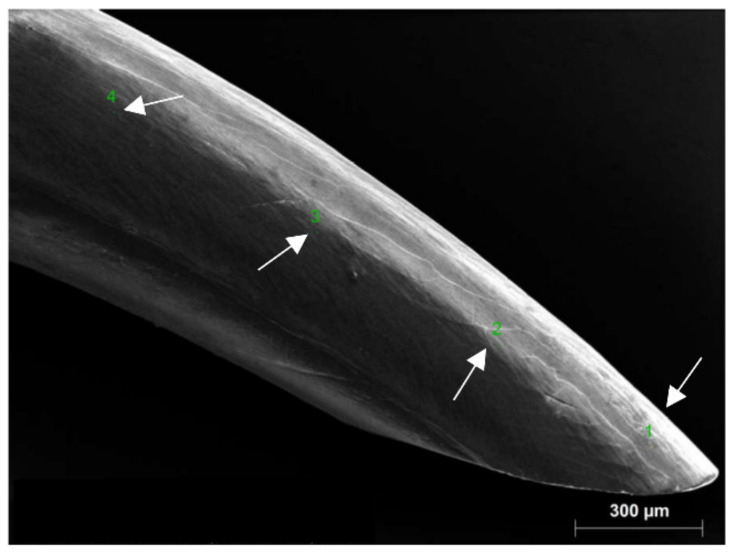
SEM image of spiny mouse incisor. Arrows indicate points in an approximate distance of 100 µm, 500 µm, 1000 µm and 1500 µm from the incisor tip at which the examination was conducted.

The examination was conducted on eight sets of incisors from spiny mice on the 0th, 2nd, 5th, 7th, 10th, 14th, 21st and 28th day of postnatal life (dpn), which were euthanised by CO_2_ inhalation (*n* = 14 for each group, *n* = 112 in total). After preparation, incisor enamel was subjected to the SEM, EDS, XRD, FTIR and Raman microspectroscope measurements to describe its surface structure, chemical composition and mechanical features. The examination was done on the labial surface at 100 µm, 500 µm, 1000 µm and 1500 µm distance from the incisor tip (Figure 1), which, as said in [35], is the region of almost completely calcified enamel. XRD and FTIR analyses were conducted at one point in the region between 100 µm to 500 µm from the incisor tip. According to [19], no sexual dimorphism is evident for *Acomys cahirinus*, thus no distinction between sexes was made in the presented research.

### 2.1. Sample Preparation

Extracted teeth were disinfected in aqueous 0.5 % Chloramine-T solution (Poch S.A., Gliwice, Poland) dissolved in deionised water at 4 °C for 24 h, washed with deionised water, and stored in a 0.9% NaCl solution at 4 °C for no longer than seven days [36,37] before the further examination.

### 2.2. SEM Imaging and EDS Measurements

Energy dispersive spectroscopy (EDS) along with scanning electron microscopy (SEM) imaging was applied to determine the elemental composition and structure of examined enamel (ZEISS Ultra Plus Field Emission Scanning Electron Microscope with Bruker X flash EDS detector, Billerica, MA, USA). To reveal the HA structure, teeth were immersed for 15 s in 35% orthophosphoric acid (H_3_PO_4_) and rinsed with deionised water (Millipore, Bedford, MA, USA) for the 20 s [38]. Samples for SEM imaging (1 for each group, *n* = 8 in total, treated with H_3_PO_4_) and EDS measurements (*n* = 16, 2 for each group, not treated with acid) were placed in a desiccator under vacuum conditions for 24 h to dry. Previous to introducing the samples to the SEM chamber, teeth were attached with carbon tape to an aluminium stage and examined with EDS (accelerating voltage 20 keV). To examine the enamel structure, samples were coated with an ultrathin gold (Au) film with sputtering ion equipment (Sputter Coater SC7620, Emitech, Fall River, MA, USA). The detector was operating in a high vacuum at an accelerating voltage of 5 keV with a working distance of 9 mm. X-ray spectra were acquired and analysed in terms of the percentage share of Ca, P, C, and Fe in the enamel structure depending on the age of the offspring and the distance from the tip of the incisor.

### 2.3. AFM Measurements and Data Analysis

Measurements were taken on *n* = 24 incisors (*n* = 3 for each group). The teeth were mounted on plates by an adhesive tape and examined under an optical microscope to determine regions next submitted to Peak Force Quantitative Nanomechanics Mapping (PFQNM) analysis. Multi-Mode 8 with a NanoScope V controller equipped with a J-type scanner (Bruker, Billerica, MA, USA) was used. Both height and elasticity modulus were mapped simultaneously. For each region, three maps sized 1 µm × 1 µm, with a spatial resolution of 512 × 512 points per image were collected. Each scan was performed with a 0.5 Hz rate. Measurements were conducted at room temperature and 40% of relative humidity. The z-range was set at 300 nm. Rectangular cantilevers 125 µm long, with a nominal tip radius of 30 nm, a nominal spring constant of 200 N/m and a resonance frequency of 525k Hz (RTESPA-525, Bruker, Billerica, MA, USA) were calibrated prior to measurements using the absolute method.

The modulus of elasticity was calculated by fitting part of the unloading curve (between 90% and 30% of the force range) using the Derjaguin–Muller–Toporov (DMT) model [39]. Histograms of the stiffness distributions were calculated separately for each scan. The maxima were located with OriginPro 2021b (OriginLab Co., Northampton, MA, USA) by fitting a linear combination of Gaussian functions [40].

Additionally, the parameters describing surface roughness (average roughness S_a_ and root mean square roughness S_q_) were determined for a height function Z_ij_ (x,y) defined over a certain XY plane, according to the Equations (1) and (2):

(1) $$S_{a}=\frac{1}{N_{x}N_{y}}\sum_{j=1}^{N_{y}}\sum_{i=1}^{N_{x}}\left|Z_{\mathrm{ij}}\right|$$

(2) $$S_{q}=\sqrt{\frac{1}{N_{x}N_{y}}\sum_{j=1}^{N_{y}}\sum_{i=1}^{N_{x}}Z_{\mathrm{ij}}^{2}}$$

where N_x_ and N_y_ are the sampling rates along the X and Y axis, respectively [41,42].

### 2.4. X-ray Diffraction

The samples (*n* = 24, 3 for each group) were measured in θ–2θ geometry (Rigaku XtaLAB diffractometer, Tokyo, Japan) with an X-ray beam diameter of 0.1 mm. The device was equipped with a MicroMax-007 HF rotated anode X-ray source and a Pilatus 300 K area detector operating over 6–110 deg with a resolution of 0.078 deg and 10 min counting time per frame. During measurement, the sample was rotated around the direction perpendicular to the θ–2θ plane (around the z-direction) for signal averaging from 0.1 mm sample slices. The obtained results were processed in CrysAlisPRO software (Rigaku, Tokyo, Japan). Based on the received diffractograms, FWHM of reflection peak from (002) plane, mean size of nanocrystallites D along with z-direction, and lattice parameters a and c of the hexagonal cell were determined.

The mean size D of the nanocrystallites was calculated according to the Scherrer equation [43] (Equation (3)):

(3) $$D=\frac{K\lambda }{{\mathrm{FWHM}}_{002}\cos\theta }$$

where D is the mean size of the ordered crystalline domains, K is a constant related to the crystallite shape (0.9), FWHM_002_ is the full width of the peak at half of the maximum intensity counting the apparatus broadening of 0.08 deg (limited by the detector resolution at 40 mm from the sample) for 002 plane, λ is the wavelength of X-ray radiation (1.5478 Å) while θ is the peak position [44,45]. Lattice spacing d_hkl_ was determined from the Bragg’s law for the Miller indices (300) and (002) according to the Equation (4):

(4) $$\lambda =2d_{\mathrm{hkl}}{\sin\theta }_{\mathrm{hkl}}$$

While lattice parameters a and c for the hexagonal unit cell were calculated from the Equation (5):

(5) $$\frac{1}{d_{\mathrm{hkl}}^{2}}=\frac{4}{3}\left(\frac{h^{2}+\mathrm{hk}+k^{2}}{a^{2}}\right)+\frac{l^{2}}{c^{2}}$$

where h, k and l are the Miller indices that are the reciprocal intercepts of the plane on the unit cell axes [45].

Bragg peaks and crystallographic planes were determined using Mercury CSD 3.10.1 software (CCDC, Cambridge, UK) from the hydroxyapatite references (No. 2300273, Crystallography Open Database, and No. 96-901-0053, High Score Plus package software). The peak position and FWHM were calculated from the Gaussian function’s fits to every peak using the Grams/AI 8.0 (Thermo Scientific, Waltham, MA, USA).

### 2.5. Raman Spectroscopy

Raman spectra for *n* = 24 samples (3 for each group) were acquired using the inVia™ Confocal Raman Microscope system (Renishaw, Edinburgh, UK). The measurements were performed at four points along the labial surface of the spiny-mouse incisor. The spectrometer was equipped with an EMCCD detection camera (Newton 970, Andor Technology, Belfast, Northern Irelan) and a 50× objective with a numerical aperture of 0.75 (Leica, N Plan EPL, Wetzlar, Germany). A He-Ne laser (Renishaw, Gloucestershire, UK) with a wavelength of 633 nm and 20 mW power was used to collect the data. The spectra were recorded in the spectral range of 146–1930 cm^−1^ at 1 cm^−1^ spectral resolution (1200 grooves/mm diffraction grating). The photochemical bleaching for 120 s was applied before Raman data acquisition to minimise tissue fluorescence. Each Raman spectra was pre-processed by cosmic ray removing and corrected by subtracting a six-order polynomial fitting curve of fluorescence backgrounds using WiRE 4.4 software (Renishaw, Wotton-under-Edge, Gloucestershire, UK).

After the acquisition of the spectra, mineral distribution was calculated from the relative evaluation of the band areas. The carbonate-to-phosphate ratio was calculated using the area under the bands associated with phosphate and carbonate components [46]. All spectra were subjected to rubber band correction (RBC) with 15 iterations before integrals were calculated using OPUS 7.5 (Bruker Optics, Ettlingen, Germany). After the deconvolution procedure, the integral regions were defined as ν_1_PO_4_^3−^ at 980–920 cm^−1^ and ν_3_ CO_3_^2−^ at 1093–1057 cm^−1^. The peak parameters were obtained by curve fitting with two Gaussian-Lorentzian curves that fit the 1030–1100 cm^−1^ region of the Raman spectrum using the OriginPro 2016b software (OriginLab Co., Northampton, MA, USA). Crystallinity was calculated as the full-width half-maximal (FWHM) bandwidth of the ν_1_PO_4_^3−^ band. The FWHM was calculated from the width of the Gaussian curve at 960 cm^−1^.

### 2.6. Attenuated Total Reflectance-Fourier Transform Infrared (ATR-FTIR) Spectroscopy

Mid-infrared absorption spectra in ATR mode were obtained using an IRSpirit (Shimadzu, Kyoto, Japan) equipped with a DLATGS detector and QATR™-S Single-Reflection ATR Accessory (Shimadzu, Kyoto, Japan). A 2 mm labial surface of spiny-mice incisor samples was transferred to a diamond ATR crystal and pressured against its surface with a swing pressure clamp mechanism to provide better contact. Spectra were collected with 36 spectral scans at a resolution of 4 cm^−1^ in the range of 4000 and 600 cm^−1^, and the ATR correction was applied. (OPUS 7.5 software, Bruker Optics GmbH, Ettlingen, Germany). The background spectra were collected from a pure crystal and subtracted automatically using the same measurement parameters. All spectra were smoothed using 9 smoothing points, baseline-corrected with the polynomial method, and normalised to the intensity at 1010 cm^−1^. The spectra were repeated three times for each experimental object (*n* = 8).

For the qualitative analysis, the absorption bands considered for this study were the superposition of the ν_1_ and ν_3_ vibration modes of phosphate and the ν_3_ stretching vibration mode of B-type carbonate [47]. The areas under the considered bands were calculated after normalisation by the phosphate band’s area of the ν_1_ and ν_3_ vibration modes [48,49]. Thus, the relations between the area of 1485–1365 cm^−1^ carbonate band and the area of phosphate band (1180–890 cm^−1^) were used to estimate the carbonate-to-phosphate ratio (C/P).

The transition from poorly crystalline hydroxyapatite into well-crystallised HP is accompanied by the progressive splitting of the in-plane bending vibration of the phosphate band at ~600 cm^−1^ (ν_4_PO_4_) and of the asymmetric stretching vibration at 1100 cm^−1^ (ν_3_PO_4_). However, Weiner and Bar-Yosef [50] proposed estimating the amount of splitting by measuring the intensities of the two symmetrical bending bands at 603 cm^−1^ and 567 cm^−1^, and 590 cm^−1^, which is the minimum between these bands after drawing a baseline from 750 to 500 cm^−1^. In this way, the crystallinity index (CI)_FTIR_ is the value calculated according to the following Equation (6):

(6) $${\mathrm{CI}}_{\mathrm{FTIR}}=\frac{A_{603}+A_{567}}{A_{590}}$$

where A_x_ is the absorbance at the given wavenumbers.

### 2.7. Data Structure and Analysis

The statistical analysis was performed using Statistica13.1 (Palo Alto, CA, USA) and Origin2021b (OriginLab, Northampton, MA, USA). One-way ANOVA or mixed ANOVA with repeated measurements was used to assess the changes in enamel properties. One-way ANOVA was applied for XRD and FTIR analyses. Mixed ANOVA with repeated measurements was conducted when the distance factor as a within-subject variable was considered (Raman microspectroscopy, AFM and EDS analyses). Normality of data distribution and variance homogeneity were checked by the Shapiro–Wilk and the Levene test, respectively. When the normality of variance or the variance homogeneity was not met, instead of a one-way ANOVA application, the data were analysed by a Kruskal–Wallis H-test and the post-hoc analysis using Dunn’s test. The assumption of sphericity was assessed using the Mauchly test. If sphericity cannot be assumed, Greenhouse–Geisser correction was calculated. The significance level p was set at 0.05. After analysis, further post-hoc tests (Tukey) were carried out to ascertain the nature of the differences among and within the groups.

### 2.8. Predictive Modelling

Artificial neural networks (ANNs) were used in relation to the purpose of our work to propose a scheme of enamel development, which could be used as a comparative material in environmental, nutritional and pharmaceutical studies. It was assumed that each developmental disturbance leads to certain fluctuations in the measured parameters and thus may indicate a different time of life. Hence, an established model aimed to predict the day of postnatal life based on collected data concerning EDS (elements concentration and Ca/P ratio according to dpn), XRD (FWHM and a-lattice parameter according to dpn), Raman and FTIR spectra (crystallinity and carbonate to phosphate ratio) and mechanical properties (DMT modulus). Data were standardised according to Equation (7).

(7) $$z_{\mathrm{ij}}=\frac{x_{\mathrm{ij}}-\bar{x}}{\sigma }$$

where: z_ij_—new variable element, x_ij_—original variable element, $\bar{x}$—a mean of the original variable, σ—original variable standard deviation. The chart was randomly divided into training, verifying, and testing and then introduced to the network input. A multilayer perceptron network (MLP) was used in the process of prediction. MLP is one of the oldest artificial neural network models but is still successfully applied today. The fundamental element of the MLP network is a neuron that is sigmoidal in nature, for which the output signal takes values from 0 to 1 for unipolar functions or from −1 to 1 for bipolar ones. MLPs are strictly organised, and neurons are arranged into three main layers: input, hidden, and output. The number of hidden layers and the number of neurons in each were determined experimentally, using the growth method [51]. The increase in the number of neurons in the hidden layer causes an increase in teaching, validation and testing rates but only to a certain point, after which they begin to fall. Simultaneously, the error values decrease to a minimum and grow after crossing the optimal point. This is due to over-learning, during which the network over-fits the learning points, accompanied by unstable behaviour for data not presented during the learning phase (the network cannot generalise) [52]. Due to that fact, networks with the maximum teaching, validation and testing rates (correlation coefficients R^2^) simultaneously having the minimum sum of squared errors (SSE) were considered optimal. Additionally, the root-mean- square error (RMSE) values were calculated. SSE informs which part of the variation of the dependent variable cannot be explained by the model. At the same time, RMSE is a measure of accuracy that explains how the data is concentrated around the regression line. The process of generating a neural model was carried out using Statistica 13.1 (Palo Alto, CA, USA).

## 3. Results

### 3.1. Surface Structure

**Figure 2:**
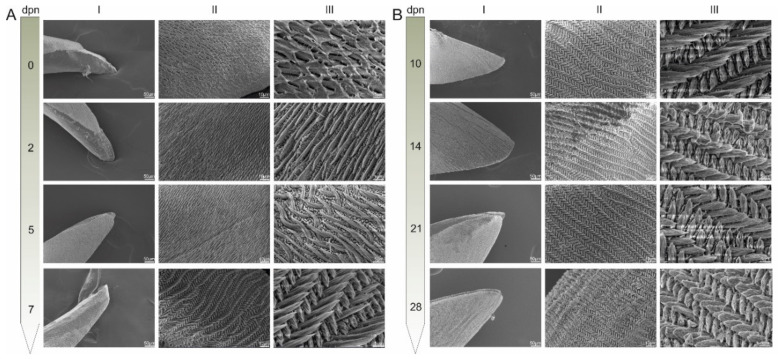
SEM images of incisor tip (I, mag. 500×) and etched enamel surface (II, mag. 2500×, III, mag. 10,000×) pictured in 0th, 2nd, 5th, 7th (**A**) and 10th, 14th, 21st and 28th (**B**) day of postnatal life (dpn).

To investigate enamel surface morphology in the micrometre and nanometre-scale and assess its mechanical features qualitatively, scanning electron microscopy (SEM) and atomic force microscopy (AFM) [1,37,53,54] were applied. SEM images, presented in Figure 2, show changes in the surface structure of incisor enamel during the period considered. It is clearly visible that along with maturation (since the 5th dpn), the outer enamel is taking the shape characteristic of rodent incisors. Long prisms are being arranged in an alternating manner due to the different angles of inclination of the prisms in successive rows, which causes them to decussate [11] and create a so-called radial enamel. Additionally, crystallites increased their number in the prism and interprism regions, which is manifested by denser structures observed, especially on the 14th, 21st and 28th dpn. Interestingly the sole tip is not covered with the enamel layer during the first two days of postnatal life (Figure 2A (I)).

**Figure 3:**
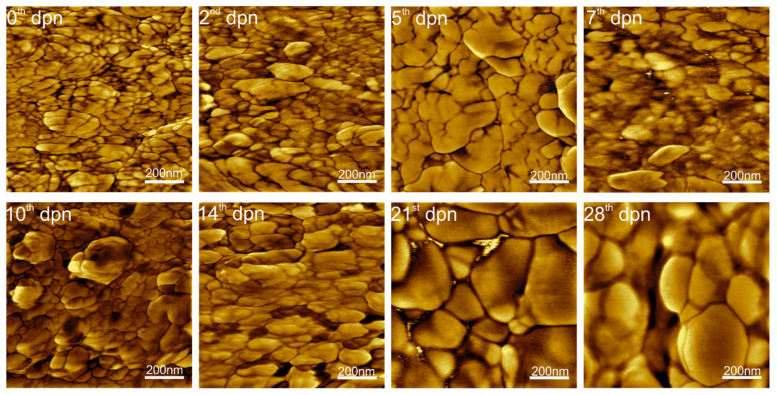
AFM scans (1 µm × 1 µm, phase) of the enamel surface of the incisors done at a distance of 500 µm from the tip for the 0th, 2nd, 5th, 7th, 10th, 14th, 21st and 28th day of postnatal life (dpn).

**Figure 4:**
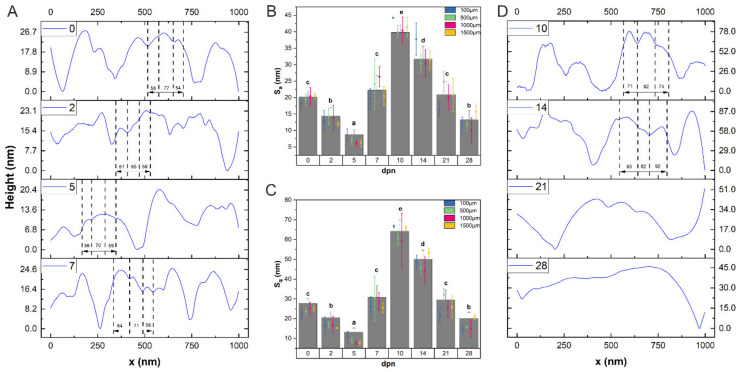
Averaged height profiles over the scanning distance of 1 µm (**A**,**D**). Average roughness S_a_ (**B**) and root mean square roughness S_q_ (**C**) with corresponding statistics according to days (columns) and distance (error bars plot). The dashed lines in the height profiles present borders between the chosen HA crystallites, which are not marked for the 21st and 28th day of postnatal life (dpn). Data are presented as mean ± SD. Different letters indicate significant differences in groups (at *p* < 0.05).

HA ribbons (HA crystallites) are roughly hexagonal in cross-sections (Figure 3), about 55–90 nm wide (Figure 4) and surrounded by a thin (about 2 nm) layer. Along with development, crystallite growth and thickening may be observed. It is worth noting that the more intense growth of HA crystals occurred between the 7th and 14th day, which is manifested by the appearance of significantly higher irregularities on the surface (Figure 4), reaching its maximum at the 10th dpn.

When the crystallites grew, they began to form larger and more regular structures, clearly visible on the 21st day of life. Since then, the crystallites adhere to each other more tightly, making spaces narrower and even undetectable, as seen in Figure 4D. The final enamel seems to be completed at about the 28th day of postnatal life, as shown in Figure 3 and Figure 4.

### 3.2. Chemical Composition

**Figure 5:**
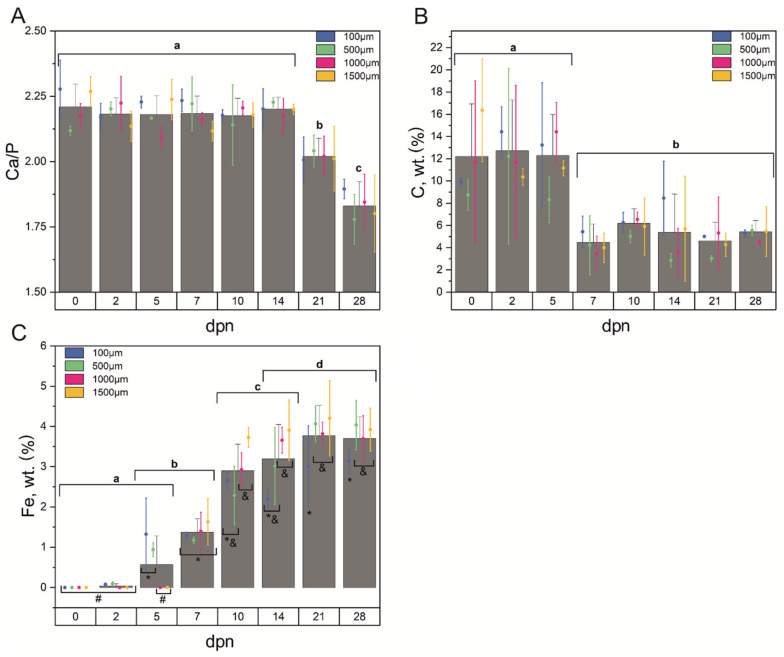
Ca/P ratio (**A**), carbon content (**B**) and iron concentration (**C**) based on EDS analysis determined for the 0th, 2nd, 5th, 7th, 10th, 14th, 21st and 28th day of postnatal life (columns) and 100 µm, 500 µm, 1000 µm and 1500 µm distance from the incisor tip (error bars plot) with corresponding statistics. Data are presented as mean ± SD. Different letters indicate significant differences in groups (at *p* < 0.05) for mean values and symbols (*, #, &) for iron concentration depending on the distance.

The enamel changes its chemical composition during maturation and under the influence of external factors. Thereupon, the energy dispersive spectroscopy (EDS) technique was applied for qualitative analysis of enamel tissue [55]. As mentioned before, the presented research was focused on the almost entirely calcified enamel region. Since the main building blocks of enamel are calcium and phosphate ions, the Ca/P ratio was determined based on EDS results. Energy dispersive spectrometry revealed that over the teeth growth Ca/P ratio remained constant until the 14th dpn. On the 21st day, a significant decrease might be observed, and the Ca/P ratio continued to fall, reaching a mean level of 1.829 on the 28th dpn (Figure 5A). Simultaneously, no significant differences according to the distance from the incisor tip were found.

The carbon content (Figure 5B) changed over the growth period, showing a higher level for the first days of life, while from the 7th dpn, a significant drop may be observed. It can also be noticed that the C content remained on a similar level from the 7th to the 28th day of postnatal life. No significant differences in C concentration over a distance from the incisor tip were found (*p* > 0.05).

According to our findings, the concentration of iron started to build into the HA structure on the 2nd day of postnatal life, increasing with tooth development and reaching the equilibrium level at around the 21st day (Figure 5C). Mixed ANOVA with repeated measurements showed that iron concentration was also significantly dependent on the dpn*distance interaction (*p* = 0.039). As shown in Figure 5C, the differences between Fe concentrations take a different shape for particular measurement points (days). The divergence starts to occur on the 2nd and more clearly on the 5th dpn when the Fe contents for the 100 µm and 500 µm distances from the incisor tip increase significantly. It is also visible that Fe cannot be detected in more distant points from the incisor tip in the initial period of life. The situation changes on the 7th dpn when Fe levels are approximately the same for all the examined points. From the 14th dpn, apart from the overall increase in the content, some stable pattern might be observed: a lower concentration of Fe at the proximity to the incisor tip (100 µm) and its higher contents in the middle (500 µm and 1000 µm) and farther (1500 µm) parts of the tooth.

### 3.3. Crystallographic Organisation

**Figure 6:**
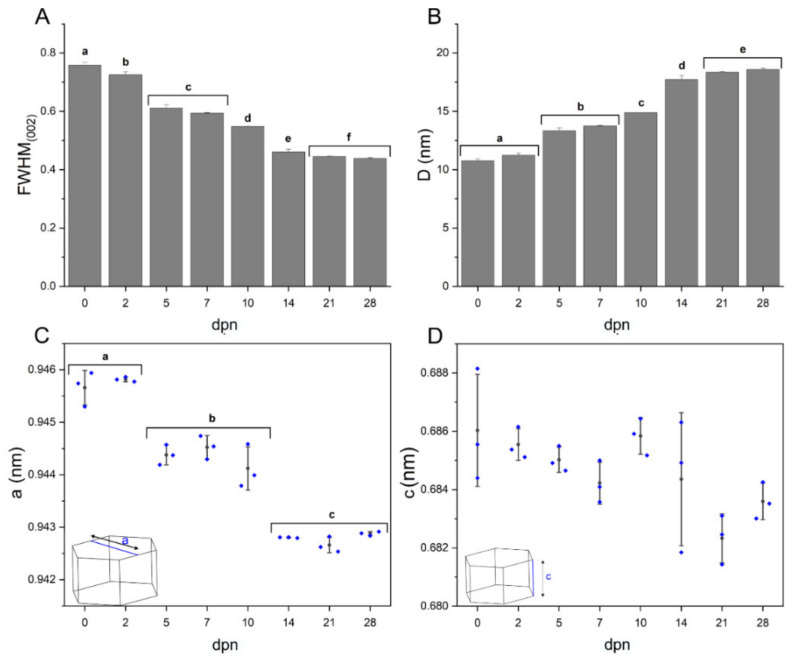
FWHM of (002) reflection peak (**A**), the mean size of HA crystallites in z crystallographic direction (**B**), lattice parameters: a (**C**) and c (**D**) calculated according to the age with corresponding standard deviations. Different letters indicate significant differences in groups (at *p* < 0.05).

An alternating chemical composition influences the spatial distribution of atoms in a hydroxyapatite crystalline phase, which can be assessed using X-ray diffraction (XRD) [56]. XRD pattern analysis and calculations based on reflection peaks at 2θ = 25.886 deg (002) and at 2θ = 32.912 deg (300) revealed that along with maturation, FWHM gradually decreased, achieving a stable level on 21–28th dpn (Figure 6A). With peak narrowing, the crystallite length (D) increased from the mean of 10.77 nm for the 0th day of life to 18.34 nm—18.59 nm for the 21st and 28th dpn, respectively (Figure 6B). The hexagonal lattice structure did not change in height (c) regarding the day of life; however, considerable changes might be registered for the a-lattice constant, which significant reduction (from the mean of 0.9457 nm to 0.9428 nm) might be observed over the considered timespan.

### 3.4. Molecular Arrangement

**Figure 7:**
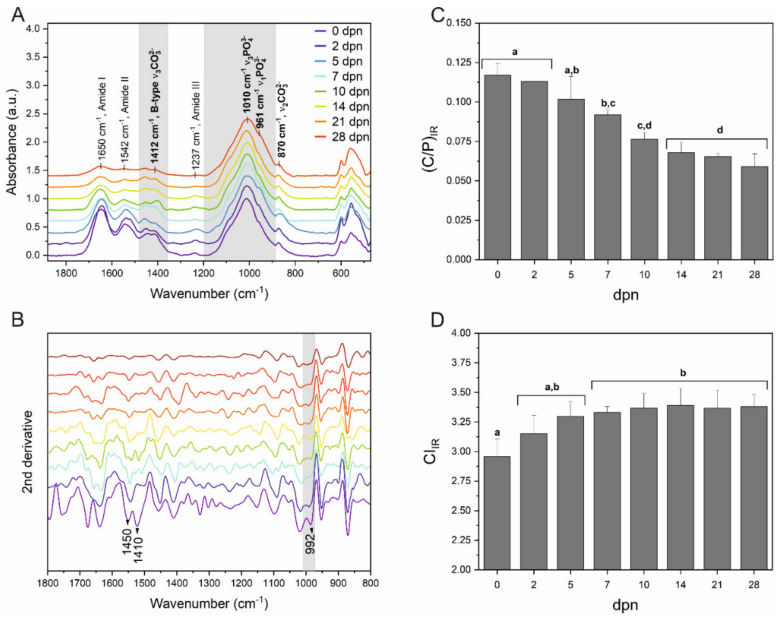
Analysis of the enamel labial surface of spiny-mice incisors by ATR-FTIR spectroscopy. (**A**) Average IR spectra in the region 460–1880 cm^−1^ of dental enamel determined for the 0th, 2nd, 5th, 7th, 10th, 14th, 21st and 28th day of postnatal life and its second derivative (**B**). Important bands for further analysis and corresponding to the phosphate (PO_4_^3−^), carbonate (CO_3_^2−^), and amide I, II and III vibrations are labelled individually. (**C**) Carbonate-to-phosphate ratio and (**D**) crystallinity index (CI) determined according to days of postnatal life (dpn). Data are presented as mean ± SD. Different letters indicate significant differences in groups (at *p* < 0.05).

Fourier transform infrared spectroscopy (FTIR) and Raman spectra collected from incisors’ labial surfaces were used to evaluate the carbonate content and crystallinity in enamel mineral composition. The ATR-FTIR spectra of the incisor samples obtained at eight stages of maturation (0th, 2nd, 5th, 7th, 10th, 14th, 21st and 28th day of postnatal life) are shown in Figure 7A. Comparing the spectra in the range of 1800–1200 cm^−1^, it was found that the bands’ intensity characterising the amide bands of enamel protein matrix showed a high level for the early stage of development and the decrease intensely after 10th dpn. All spectra exhibited similar spectral characteristics in the region between 1200 and 460 cm^−1^, except for a shoulder at 961 cm^−1^ that emerged after 2nd dpn. The phosphate bands are identified by vibrational mode arising from the phosphate group at 961 cm^−1^ (ν_1_PO_4_ stretching mode) and a strong and broad band at ∼1012 cm^−1^ (ν_3_PO_4_^3−^) [57].

The second derivative of the spectrum was used for a more detailed analysis due to the risk of overlapping bands [58,59], as shown in Figure 7B. The most distinguishing changes in the second derivative spectra are related to loss in the intensity of the amide I and amide II regions and the bands assigned to the B-type carbonate substitution detected at 1410, 1450 cm^−1,^ especially after 10th dpn. Furthermore, the intensities of the bands in the range of 900–1100 cm^−1^ have significantly reduced from the younger to older teeth samples (Figure 7B).

The substantial changes in the mineral phase of the postnatal spiny-mouse incisor can be identified by the carbonate-to-phosphate ratio. The relative carbonate content was estimated from the ratios of the areas under the ν_3_CO_3_^2−^ modes to the ν_1_PO_4_. Figure 7C presents the distribution of the C/P as a function of postnatal age. A statistically significant difference was observed from 0th dpn to 10th dpn (*p* < 0.05). Interestingly, a gradual decreasing carbonate content trend was observed until the 14th dpn, after which it maintained a constant level. However, the crystallinity index did not show significant differences between the 5th and 28th dpn and exceeded 3.25 after the 5th dpn, indicating enamel maturation at the same level (Figure 7D).

**Figure 8:**
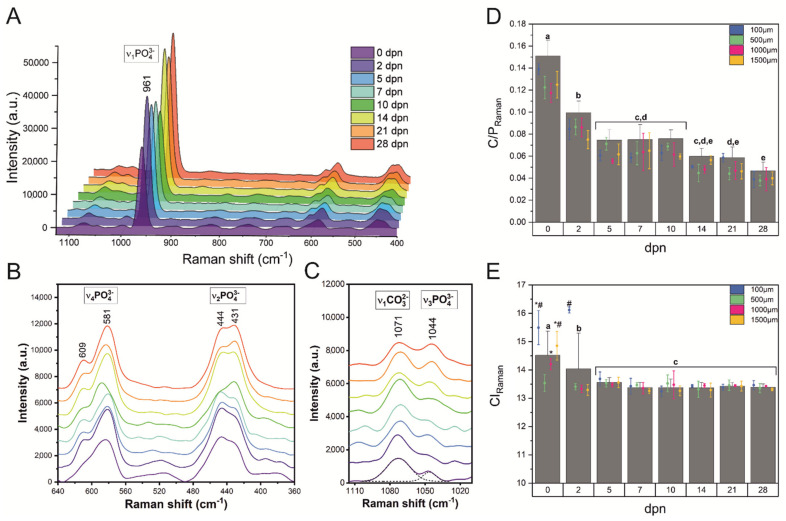
Raman spectra of the selected point along the spiny-mice incisor labial surface in the different stages of maturation (**A**), the spectral region of the ν_2_, ν_4_ phosphate group (**B**) and the spectral region of the ν_3_ phosphate and the ν_1_ B-type carbonate groups (**C**). (**D**) The carbonate-to-phosphate ratio expressed as the area of the 1070 cm^−1^ divided by the area of 960 cm^−1^ (C/P). The band parameters were obtained by fitting the two Gaussian-Lorentzian curves in the region of 1030–1100 cm^−1^ (shown only a sample fitting). (**E**) The crystallinity index expressed as the FWHM of 960 cm^−1^ (CI) obtained for the four measurements sites collected from 100, 500, 1000 and 1500 μm distance from the tip of the incisor. (**D**,**E**) were prepared in relation to days of postnatal life (dpn; columns) and distance from the incisor tip (error bars plot) and are presented with corresponding statistics as mean ± SD. Different letters indicate significant differences in groups (at *p* < 0.05), and symbols (*, #) for CI_Raman_ values depending on the distance.

Raman μ-spectroscopy was also used to obtain information on the mineral composition of the incisor at 0−28 days of postnatal life at four points on the labial surface of the tooth (Figure 1). Because there were no significant differences between the measurement points, Raman spectra are shown in Figure 8A only as a function of postnatal age.

A typical Raman enamel spectrum is characterised by the region of the strong ν_1_(PO_4_)^3−^ vibration located at 961 cm^−1^, well-separated bands assigned to the ν_2_(PO_4_)^3−^ and ν_4_(PO_4_)^3−^ bending modes at ~430 and 580 cm^−1^, respectively [27,28]. The band located at ~1070 cm^−1^ is a superposition of the ν_1_ of B-type carbonate with carbonate substituting for phosphate, and the ν_3_ of phosphate (~1040 cm^−1^) [60]. It can be seen that the bands related to mineral content, including the ν_1_ carbonate vibration and ν_1_ and ν_2_ phosphate modes, exhibit the most prominent spectral changes. The phosphate bands at 961 and 1044 cm^−1^ especially show variable signal intensities and their intensities increase depending on age, while the intensity of 1071 cm^−1^ decreases (Figure 8A,C). Note that when comparing 14th, 21st and 28th dpn with the younger enamel samples, we can also see a redistribution of band intensities in the region of ν_2_(PO_4_)^3−^ bending modes.

The carbonate-to-phosphate ratio and the crystallinity index characterised by the FWHM of ν_1_ mode are poorly correlated with age (Figure 8D,E). By considering the carbonate-to-phosphate ratio in all the analysed groups, the highest value was found in 0th and 2nd dpn (*p* < 0.05), while the lowest ones were displayed in the enamel of older incisor (14th, 21st and 28th dpn, *p* < 0.05) (Figure 8D). The observed fall in carbonate content reflects the increase in the crystallinity observed in the XRD study manifested by a gradual decrease in FWHM002 (Figure 6A). On the other hand, 5th to 28th dpn showed no significant differences (*p* > 0.05) in the case of CI index.

### 3.5. Mechanical Features

**Figure 9:**
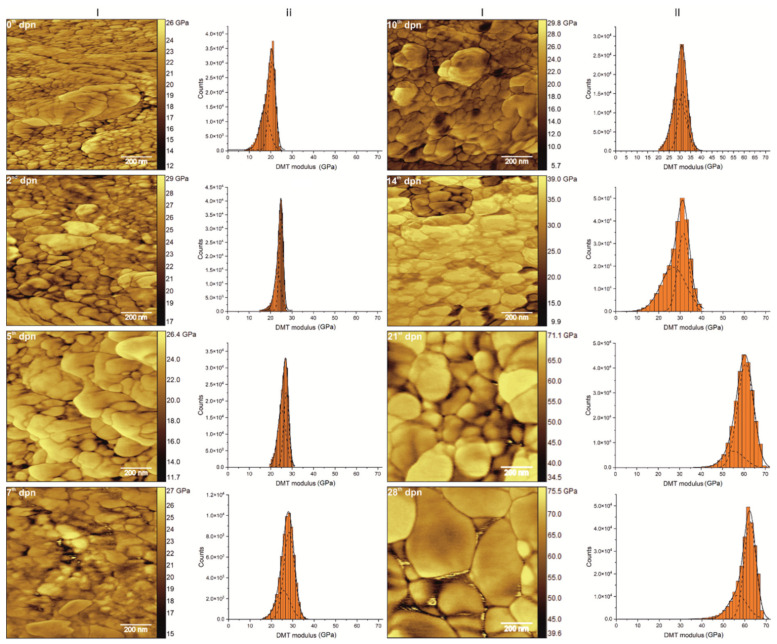
Peak Force QNM representative scans (1 µm × 1 µm) of enamel surface (I) with corresponding histograms of averaged DMT modulus distribution (II) determined for 0th, 2nd, 5th, 7th, 10th, 14th, 21st and 28th day of postnatal life (dpn) with Gaussian functions fitted. Scale bars are equal to 200 nm.

AFM scans of outer enamel in the elasticity domain (Figure 9 (I)) revealed a dual structure of the surface: hydroxyapatite crystallites bonded by the organic matter [61]. Therefore, as shown in Figure 9 (II), the distribution of the elasticity modulus is not uniform over the whole scanned area and can be separated into two closely-related groups. The first peak is notably broader, with a lower modulus and lower intensity. The second one is significantly narrower and characterised by higher modulus values and higher intensities.

**Figure 10:**
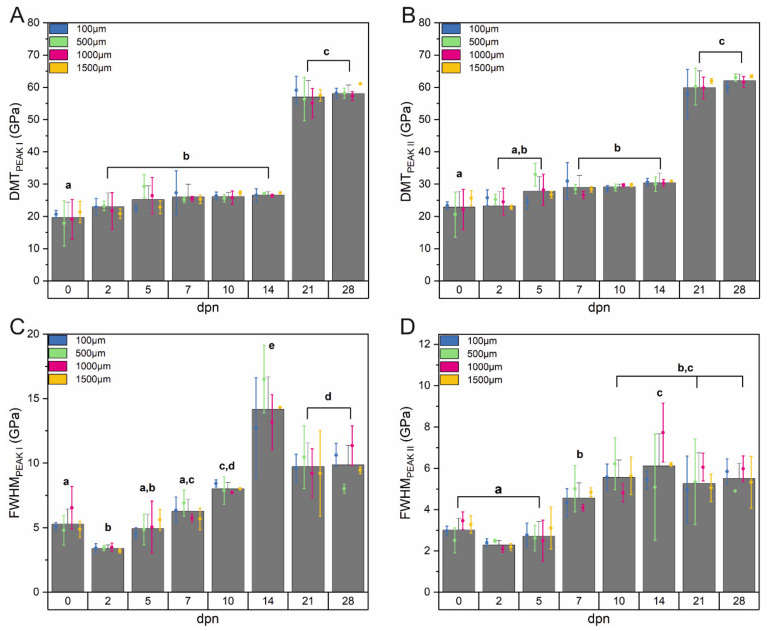
The average values of DMT modulus and FWHM according to the day (columns) and the distance (error bars plot) with corresponding statistics for peak I (**A**,**C**) and peak II (**B**,**D**). Data are presented as mean ± SD. Different letters indicate significant differences in groups (at *p* < 0.05).

For both observed peaks, the DMT modulus grew instantly along with maturation (Figure 10A,B) at a similar pace. Since the 0th dpn, a slight increase in DMT value can be registered. The increase continues until the 5th dpn, when it reaches a steady level and remains almost unchanged for the following days, up to the 14th dpn. Although the mean values of the DMT modulus do not change substantially between 5th and 14th dpn, a significant broadening of the peaks and a slight shift of the modulus ranges towards higher values might be observed in this period. It should be noted that the full width at half maximum, after the initial drop on the second day of postnatal life, started to grow, reaching its maximum in the 14th dpn (Figure 10C,D). On the 21st and 28th day of life, a significant increase (for about 25–30 GPa) in the value of the modules can be registered. Simultaneously during that period, FWHM stabilised at a mean level of 9.8 GPa for the first and 5.4 GPa for the second peak.

It is worth mentioning that the difference between the maxima of determined peaks is in the range of 2–4 GPa, irrespective of time. Calculations showed no statistical difference between the values of DMT modulus and FWHM depending on the distance from the incisor tip (Figure 10).

### 3.6. Regression Model

**Figure 11:**
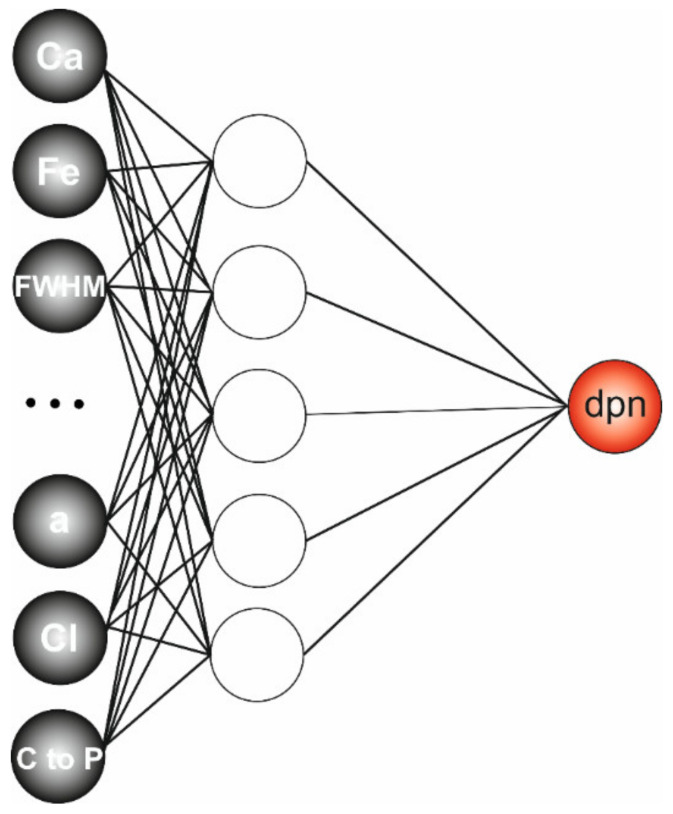
Multilayer perceptron network used in the process of prediction.

In order to present a complete study, a computational regression model whose task was to predict the age of the enamel based on information about its broadly understood structure was built. The proposed model is based on artificial neural networks (ANNs), which are computational models regarded as intelligent systems used to solve complex problems with nonlinear relationships using many independent parameters [52,62,63,64]. MLP network with 13 inputs, one hidden layer and one output (Figure 11) was used to predict the day of postnatal life based on collected data. The network was trained with the Broyden–Fletcher–Goldfarb–Shannon (BFGS) algorithm [52] for 100 epochs. The training quality and relative errors for different responses during training, validation and testing are presented in Table 1.

The correlation coefficient, which measures the relationship between experimental and predicted data, takes values close to one, indicating a good model fit. It is also proved by the relatively low levels of SSE and RMSE errors.

To assess the relationship and the influence of each input parameter on the predicted output, a sensitivity analysis was conducted. During such an analysis, the ratio is calculated from the relation of an error obtained for the data set without a single variable and the error obtained with a complete set of variables. The greater the error after the variable rejection, the more sensitive the network is to the lack of this variable. Conversely, a ratio close to or less than one indicates that the rejection of the variable has no effect or improves the network quality. The conducted analysis revealed (Table 2) the less important variables: crystallinity determined from Raman and FTIR spectra, carbon and calcium content. Mentioned variables were then removed from the model, and the new network, with nine inputs, five neurons in the hidden layer and one output, was taught on the remaining ones. The results achieved were slightly better, especially considering errors in training and testing, as shown in Table 3.

The constructed MLP network was then used to predict dpn using data not previously applied in the training process. MLP 9-5-1 unmistakably recognized the dpn after giving the nine input parameters (see Table S1 in Supplementary Materials).

## 4. Discussion

The constant growth of incisors and clear recognition of all space-time stages during amelogenesis [65] allow them to be used as an experimental model of general tissue growth processes and cellular interactions at all stages of enamel development [2,3,13]. The presented study proposes the spiny-mouse incisor as a model system to track the mineral composition and structure alterations during the postnatal period, from the 0th to the 28th day of life.

The initiation of enamel formation in rodent incisors begins in the neonatal period in the apical zone, where enamel matrix proteins are secreted and develop a network for mineral deposition. Then, in the early stage, enamel ribbons in the form of amorphous calcium phosphate are located in the protein-rich matrix. Next, rod and interred regions are formed in the inner and outer enamel. The following step is the mineralisation stage, during which the ameloblasts secrete proteinases such as metalloproteinase (MMP20) and kallikrein 4 (KLK4) [10,66]. Those proteinases are responsible for the degradation of the organic matrix and the reduction of matrix content. During the late maturation stage, originated products of matrix proteins accumulate within the enamel [67,68] to form a substance that establishes the formation of crystallites’ shape, size, and orientation. As a result, ACP crystallites grow in thickness and width and subsequently are transformed into hydroxyapatite [4]. Then, the process of amelogenesis ends, usually prior to eruption, with the complete mineralisation of the enamel [6].

Observing the *Acomys* incisors enamel surface in the region of theoretically fully mature tissue revealed the hexagonal structures’ presence, with a diameter increasing from 50 nm to about 90 nm and length between 10.62 nm for young animals to 18.70 nm for 28 days old ones. According to the literature, the resulting HA crystallites are long, roughly hexagonal structures (from several to several hundred nm depending on tooth and species), approximately 60–70 nm in diameter, built with single units stacked on top of another [53,61,69]. The hexagonal shape of the crystallites is lost during the final phase of their growth as they press against each other and become irregular in shape [70]. Crystallites are surrounded (or glued) by the organic-derived substance [71]. Previous studies indicated that these are mainly proteins, especially amelogenins [12,72]. The latest findings of Gordon et al. [73,74] complemented earlier observations and showed that single nanowires are separated by grain boundaries—flat interfaces between two adjacent enamel crystallites (simple) or among at least three crystallites (multiply). The authors pointed out that they take the form of Mg-substituted amorphous calcium phosphate, including organics, carbonate, and possibly water.

As shown on the AFM scans of the outer enamel (Figure 3), HA nanowires are surrounded by a thin (about 2 nm) layer of a substance with slightly different properties, indicated by the not uniformly distributed DMT modulus. The outer enamel layer is the last to be formed, so the presence of organic remains and not fully crystalised phase, representing mineralized replacements of original matrix-mineral structures among crystallites, is entirely possible [4,75], especially in the first days of life. After the 14th dpn, crystallites started to form larger structures. Some of the spaces between the crystals have become blurred, meaning they have been filled with the same material that the crystallites are made of. Simultaneously the decrease in surface roughness, evident for the 21st and 28th dpn, may be observed. It is probably connected with the transformation of the stabilized amorphous mineral into the crystalline phase [76] and space-building by mineralized tissue. The transfiguration registered by FTIR spectroscopy as the reduction of intensities in the amide regions and the substitution of bands assigned to the B-type carbonate was mainly observed since the 14th dpn. The above findings are in line with other observations (e.g., narrowing of the first peak in DMT distribution) and suggest a slight change in the composition of the material found in the boundaries; however, most of it remains in its previous form.

Mature enamel consists on average of 95% hydroxyapatite (HA) crystals (Ca_10_(PO_4_)_6_(OH)_2_), 4% water and 1% organic matter [1]. Calcium and phosphate ions, the main building blocks of enamel, enter the tissue to facilitate crystal growth during maturation [69]. Although their concentrations vary independently, the relative content of Ca and P is critical for sustaining mineral homeostasis and tissue metabolism. Their co-dependence is evident in teeth growth and development [77]. For the ideal hydroxyapatite composition, the Ca/P molar ratio is 1.67; however, natural HA observed in teeth can have different substitutes for Ca^2+^ (Na^+^, K^+^, Mg^2+^) and phosphate and hydroxyl (CO_3_^2−^, F^−^, HPO_4_^2−^, Cl^−^ and H_2_O) in comparison to those observed in HA stoichiometric form [45,78]. Therefore the Ca/P ratio may vary within limits from 1.33 to 2.0 [79]. The resented research showed that the Ca/P ratio was stable over the tested length until the 14th dpn and equal to 2.18, after which its value decreased to 1.85 for the 28th day of postnatal life. Registered amounts are highly comparable to those provided in the literature. For example, in [80], it was established that the Ca/P ratio for the wild type of mice was equal to 1.49 wt.% for the secretion stage, 1.53 wt.% for mid maturation and 1.57 wt.% for late maturation. It is known that during the maturation stage, calcium and phosphate ions uptake increase, and crystal growth accelerates until the full maturation [69,81]. However, as mentioned above, the outer edge is the last part of the tissue to mature and often erupts before completing [69]. Hence, the observed alterations in the Ca/P ratio might be associated with incomplete enamel maturity, which is also proved by FTIR spectroscopy.

Firstly, there was a time-dependent reduction in the amide bands related to eliminating most of the remaining organic matrix by specific enzymes before the HA crystallization progressed (Figure 7A). The loss in the band’s intensity in the region of 900–1100 cm^−1^ region may suggest a transition from a disordered mineral to a non-stoichiometric crystalline apatite phase, as proposed by Beniash et al. [82]. They showed that the first mineral phase formed in the freshly formed enamel could be amorphous calcium phosphate (ACP), which finally transforms into apatitic crystals [76]. Querido et al. reported that ACP could be directly identified by a band at 992 cm^−1^, and it can be a specific marker related to the ACP component of the samples [76]. In all teeth samples, the hypothetical ACP component peak at 992 cm^−1^ lost its intensity for the 21st and 28th dpn (Figure 7B). However, it is vital to keep in mind that the “ACP precursor theory” postulates that the initial mineral in enamel is amorphous calcium phosphate, which still remains controversial. On the other hand, the characteristic changes concerning this transformation were no longer as pronounced in the Raman spectra; no significant shift and narrowing of the ν_1_(PO_4_)^3−^ band position were observed [83] (Figure 8A).

FTIR and Raman spectroscopy serves as complementary vibrational techniques among various techniques applied in enamel examination [84,85,86,87]. However, due to different physical selection rules in these methods, band intensities can differ from each other [88]. In this way, FTIR spectroscopy provides information about apatite structure and amorphous phase, while the Raman spectroscopy assesses the amorphous component [76]. The results of the Raman analysis revealed only the age-dependent redistribution of the bands in the phosphate region. The bands at 431 cm^−1^ and 1044 cm^−1^ increased while the intensities of 431 cm^−1^ and 1071 cm^−1^ decreased after 14 dpn (Figure 8B,C). According to previously reported data [87,89], it may be caused by a different orientation of HA crystals in enamel rods. On the other hand, in analysing the Ca and P values (Table S2, Supplementary Materials), it might be registered that both elements increased their amount over the observed period; however, the growth dynamics of Ca are higher compared to P (1.29 to 0.95, respectively). In the form of the Ca^2+^ and PO_4_^3−^ ions, both elements can move quickly (within minutes) from blood circulation into enamel [90]. In both cases, the inclusion of these elements is heightened during the maturation stage. Since it was proved that ion transportation is bidirectional, and phosphate, for example, can diffuse out again [81], the observed alterations in the Ca/P ratio may be the result of the discussed process.

As previously noted, natural HA is quite distinct from the pure one; however, the main difference between the HA model and biological apatite is the presence of significant amounts of carbonate ions [91]. Carbon, both organic and inorganic, in the form of carbonate ions (CO_3_^2−^), may occur in the hydroxyl and phosphate positions in the HA structure. Its content is mainly related to the maturation process [86] and decreases as the enamel matures. In [92], it was shown that the total carbon level ranged from 2.36 g/100 g for human enamel to 9.78 g/100 g for ovine enamel. The presented research showed a substantial decrease in carbon content between the 0th and 5th dpn. Since then, the average carbon content was stable from the 7th to 28th day of life and is equal to 4.99 wt.%. Changes in carbon concentration were also observed as a decline in the intensities of bands assigned to B-type carbonate after the 10th day of life visible in FTIR spectra (Figure 7C) and the reduction in the relative carbonate content determined from Raman spectra (between 2nd and 10th dpn, Figure 8D). Additionally, both techniques confirmed the gradual decrease in the C/P ratio in enamel with postnatal age, although different spectral regions were analysed.

During the maturation stage, ameloblasts of rodents’ incisors incorporate iron, whereby a yellowish pigmentation might be observed. The iron is supplied through the bloodstream and deposited on the labial surface in the region of mature enamel [93,94]. Research has shown that iron-containing pigment accumulates in the form of ferritin particles [93,94] and amorphous iron-calcium phosphate [73], presumably as a result of the process of lysosomal digestion of ferritin [95], rendering enamel harder and more resistant [73]. The presented experiment revealed that Fe concentration in the region of mature enamel is distance- and time-dependent—the amount of iron changed with a distance from an incisor tip and over the first ten days of an examined period. In ameloblasts, iron is incorporated into the cells at the very early maturation stage [93] and shows a gradual increase in its level with an increase in tissue maturity [94]. Although the region where presented analyses were conducted is considered a zone of mature enamel, incisors often erupt before completing maturation [69], which might explain why iron was not detected in the first two days of life. Changes in time and distance can also be related to the known relationship between the amounts of Ca and Fe. In [87,96], it was shown that the iron concentration is inversely related to the calcium level. As might be seen in Figure S1 (Supplementary Materials), the amount of Ca is a little bit lower at a 100 µm distance from the incisor tip compared to the remaining ones, which may imply existence of “free space” for Fe ions. Additionally, it is suggested that iron and calcium might be a substitute for each other in the hydroxyapatite structure [94].

The HA crystal comprises several unit cells stacked in multiples along all possible axes and determines the crystal shape [97]. As the HA formula suggests, it consists of Ca^2+^ ions surrounded by PO_4_^3−^ and OH^−^ ions. The unit cell is built with two triangular prismatic subcells forming a rhombic prism with vertical sides, as shown in Figure S2 (Supplementary Materials). Hexagonal lattice dimensions for HA are equal to a = b = 9.432 Å and c = 6.881 Å [98]. The conducted research showed that while the cell size in the c axis did not change and its mean value was 6.846 Å, in the case of a-lattice, its value decreased with age. Since the hexagonal structure of HA can incorporate a great variety of cations and anions, the effect of a-dimension reduction might be connected with the substitution of Ca^2+^ ions by Fe^2+^/Fe^3+^ cations during enamel maturation. It was reported in numerous research [99,100,101,102], that upon Fe substitution, a decrease in HA lattice parameters a and c were observed, which was explained by the smaller ionic radius of substituting Fe^2+^ and Fe^3+^ (0.77 Å and 0.64 Å, respectively) compared to Ca^2+^ ions (0.99 Å). It should also be noted that there are studies showing an increase in the a-parameter in the presence of iron ions [103,104]; however, this effect is not fully explained.

The structure, mineral, and organic composition of enamel are proved to be strongly related to its mechanical parameters [73,105,106,107,108], which vary within tooth type, measurement method used, or the location of measurements on the tooth surface. Generally, values between 60–120 GPa are observed for Young’s modulus, while hardness covers the range between 3–5 GPa [109,110]. For example, for the wild type of mouse, the Young’s modulus determined for the lower-left mandibles was equal to 87 (±3) GPa while the hardness was equal to 3.1 (±0.3) GPa [111]. The enamel of rat incisors was assessed for the value of Young modulus related to the location on its surface [112]. It was shown to vary from 80 to 100 GPa in the upper incisor and from 76 to 92 GPa in the lower incisor. The measurements of the nanomechanical properties in human dental enamel associated with microstructural locations [113] showed that the enamel prism has a higher elastic modulus (E_prism_ = 101.7 ± 6 GPa) than the sheath region (E_sheath_ = 94.6 ± 5.6 GPa) surrounding the prism. It should be remembered that enamel is characterised by a hierarchical arrangement on several levels [71], for which mechanical properties decrease with increasing hierarchical dimension.

According to Koenigswald and Clemen [114], five levels might be distinguished: the 0 level constituted by a single hydroxyapatite (HAP) crystallite, level 1, which is a group of crystallites, level 2 formed by multiply groups of crystallites and called prisms, level 3 multiply prisms and finally level 4 which is defined by enamel type. The mineral part at each level is enveloped by a thin layer of the soft phase (intercrystallite proteins) [71], which are not incorporated within the crystalline HA. In agreement with the above classification, the elastic modulus measurements were conducted at level 1, i.e., on a group of crystallites glued with a substance of organic origin. The Young’s modulus obtained in our research showed changes in the mean values between 20–60 GPa depending on the age of the tested enamel, additionally showing an inhomogeneity connected with the presence of the organic-derived substance in its structure. The elastic modulus values measured on that level by micro-cantilever bending experiments showed 80 GPa [71] for human bulk enamel and 54 GPa for the bovine enamel [115].

The stage of enamel development is directly connected to its chemical composition, e.g., carbonates content, which was shown to decrease along with maturation [116] or iron concentration in rodent incisors which hardens the tissue. In [117], it was shown that as carbonate content increased, there was an associated decrease in crystallinity and both of these changes correlated with decreased modulus and hardness. Iron, built in the enamel structure, directly impacts carbonate type A—the higher the Fe concentration, the lower levels of A-type carbonate, so logically the more mature enamel, which resists higher loads [108]. Apart from that, it is also known that trace elements such as cadmium or titanium [104,118] influence HA crystallites’ structure and change their mechanical properties, such as shown in [119], where an increase in pigmented enamel hardness was observed.

Teeth are considered a reliable biological marker as each stage of their development is well recognised and described [11,26]. Furthermore, teeth are highly durable, resistant and relatively sensitive to external factors [32,69,105,106,118,120], making them a suitable model in medical and environmental studies. To simplify the research process, statistical models are now often used. These models, created on the basis of a selected data set, are often constructed to predict or classify [121,122,123,124] collected data. In the presented work, the information reflecting the structure of the enamel was used to develop a model estimating the day of postnatal life. However, the non-specified relationship between the input and the output requires more flexible and self-adaptive tools to consider different types of nonlinear behaviour [122]. Therefore artificial neural networks that were recently shown to be more accurate for age prediction than simple regression models [121,125] were applied. ANNs showed to be highly relevant to solving the task showing a high correlation coefficient and a relatively low error in the training course, of 0.912 for the test set. For example, in [126], where ANNs were used to estimate age through the pulp-to-tooth ratio in canines, the test set’s root mean square error was equal to 4.40. Other research aimed to understand the relationship between the composition and mechanical behaviour of ageing enamel [127]. In the mentioned work, nanoindentation and Raman spectroscopy data were used to train the Self-Organizing Maps (SOMs). The achieved results showed that applied methods could reveal complicated structure-property relationships in tooth tissues and help to understand enamel ageing and its consequences. Interestingly it was discovered that crystallinity and carbonate-to-phosphate have a strong positive correlation with the mechanical properties of the enamel and estimated age.

In our research, CI determined based on Raman spectra showed insignificant in the regression model compared to the C/P ratio. The correlation coefficients shown in Table S3 (Supplementary Materials) revealed the lowest relation between dpn and Ca, CI_Raman_, CI_FTIR_ and C. Additionally, both crystallinity indices have a weak correlation to the DMT modules, influencing their usefulness. Nevertheless, this model indicates dpn with a reasonably high accuracy, which may be a reference point for medical or environmental research.

## 5. Conclusions

The final structure of the enamel reflects the disturbances presented during development, which are manifested as changes in the morphology, chemical composition, structure, and mechanical properties of the tissue. Therefore, a multidisciplinary approach, combining SEM, EDS, AFM, and structural studies using XRD and vibrational spectroscopy, was applied to assess the age-related changes in the enamel of *Acomys* incisors during the first four weeks of postnatal life. The findings revealed that alterations covered all aspects of the structure of the examined samples and provided the basis for building a model predicting the day of postnatal life. In addition, the information obtained on morphology and structure among the analysed tooth samples extends the existing knowledge on enamel development that could be utilized as a comparative material in environmental, nutritional, and pharmaceutical research.

## Figures and Tables

**Table 1:** Results of teaching, validation and testing for the prediction with MLP network.

Network MLP 13-5-1	Training	Validation	Testing
R^2^	0.995	0.997	0.984
SSE	11.973	6.228	21.916
RMSE	0.959	0.692	1.298

R^2^—correlation coefficient, SSE—the sum of squared errors, RMSE—root mean square error.

**Table 2:** Sensitivity analysis of inputs used in MLP network training.

Independent Variable	Sensitivity Ratio	Independent Variable	Sensitivity Ratio
DMTII	7.22	Fe	1.35
Ca/P	5.46	P	1.31
FWHM002	2.63	CI_FTIR_	1.07
DMTI	1.76	C	1.06
A lattice	1.57	Ca	0.996
(C/P)_FTIR_	1.53	CI_Raman_	0.994
(C/P)_Raman_	1.49		

**Table 3:** Results of teaching, validation, and testing for the prediction with MLP network.

Network MLP 9-5-1	Training	Validation	Testing
R^2^	0.999	0.999	0.995
SSE	4.821	2.604	7.497
RMSE	0.731	0.538	0.912

R^2^—correlation coefficient, SSE—the sum of squared errors, RMSE—root mean square error.

## Data Availability

The data presented in this study are available on request from the corresponding authors.

## Supplementary Materials

The following supporting information can be downloaded at: https://www.mdpi.com/article/10.3390/ma15113993/s1, Figure S1: Dependence on Ca amount on the day of postnatal life and distance from the incisor’s tip, Figure S2: The chemical structure of synthetic hydroxyapatite with the selected cell and signed lattice parameters (Mercury 3.10.1); Table S1: The MLP prediction for new data, Table S2: Mean Ca and P concentrations according to the day of postnatal life (dpn), Table S3: Pearson’s correlation coefficients between variables used in the MLP training.
