# Supplementary material for: Surface and Structural Studies of Age-Related Changes in Dental Enamel: An Animal Model
Source: Materials (Basel). 2022 Jun 3;15(11):3993. doi: 10.3390/ma15113993 (PMC9182525; doi:10.3390/ma15113993)
Supplement: Supplementary file 1 [file materials-15-03993-s001.zip › materials-1721822-supplementary.pdf]

Supplementary Material

Surface and Structural Studies of Age-Related Changes in Dental Enamel: An Animal Development Model

Izabela Świetlicka [1*], Ewa Tomaszewska [2], Siemowit Muszyński [1], Michał Świetlicki [3], Tomasz Skrzypek [4], Wojciech Grudziński [5], Wiesław I. Gruszecki [5], Daniel Kamiński [6], Marta Arczewska [1*]

[1] Department of Biophysics, Faculty of Environmental Biology, University of Life Sciences, Akademicka 13, 20-033 Lublin, Poland; izabela.swietlicka@up.lublin.pl (I.Ś.), marta.arczewska@up.lublin.pl (M.A.), siemowit.muszynski@up.lublin.pl (S.M)

[2] Department of Animal Physiology, Faculty of Veterinary Medicine, University of Life Sciences, Akademicka 12, 20-033 Lublin, Poland; ewarst@interia.pl (E.T.)

[3] Department of Applied Physics, Faculty of Mechanical Engineering, Lublin University of Technology, 20-618 Lublin, Poland; m.swietlicki@pollub.pl (M.Ś.)

[4] Laboratory of Confocal and Electron Microscopy, Centre for Interdisciplinary Research, Faculty of Science and Health, John Paul II Catholic University of Lublin, Konstantynów 1J, 20-708 Lublin, Poland; tomasz.skrzypek@kul.pl (T.S.)

[5] Department of Biophysics, Institute of Physics, Maria Curie-Sklodowska University, Maria Sklodowska-Curie 1, Lublin, 20-031 Poland; wojciech.grudzinski@mail.umcs.pl (W.G.), wieslaw.gruszecki@mail.umcs.pl (W.I.G.)

[6] Department of Crystallography, Faculty of Chemistry, Maria Curie-Sklodowska University, Maria Sklodowska-Curie 2, 20-031 Lublin, Poland; daniel.kaminski@umcs.pl (D.K.)

* Correspondence: izabela.swietlicka@up.lublin.pl (I.Ś.), marta.arczewska@up.lublin.pl (M.A.)

Table S1. The MLP prediction for new data.

Day expected	Day predicted	P	Ca/P	Fe	FWHM	$a_{lattice}$	C/P_{IR}	C/P_{Raman}	DMT_I	DMT_{II}
0	-0,12066	11,34	2,35626	0,00	0,76786	0,94530	0,119	0,14087	21,46	24,04
2	1,87859	12,95	2,13205	0,05	0,71542	0,94586	0,113	0,09267	23,22	27,53
5	5,18481	11,95	2,24393	0,62	0,62244	0,94457	0,112	0,05944	23,42	29,22
7	6,46094	12,09	2,20265	0,94	0,59539	0,94429	0,091	0,05814	28,01	29,58
10	9,90074	15,37	2,16109	2,53	0,54813	0,94399	0,081	0,04769	27,22	29,25
14	13,80436	15,50	2,21484	3,58	0,45768	0,94279	0,060	0,04992	27,29	28,99
21	23,80931	16,98	1,94312	3,28	0,44474	0,94282	0,065	0,04434	56,85	59,95
28	28,10221	18,65	1,92130	3,8	0,43876	0,94288	0,055	0,04581	57,09	62,22

Table S2. Mean Ca and P concentrations according to the day of postnatal life (dpn).

Data	dpn	Mean amount [wt., %]	Standard Deviation
Ca	0	23.523	5.946
	2	22.239	5.851
	5	25.073	2.792
	7	27.098	5.758
	10	34.336	1.536
	14	33.209	1.768
	21	33.076	2.894
	28	33.799	1.223
P	0	10.895	2.781
	2	10.575	2.543
	5	11.364	1.117
	7	12.838	2.191
	10	15.275	0.778
	14	15.083	0.543
	21	16.411	1.475
	28	18.518	1.219

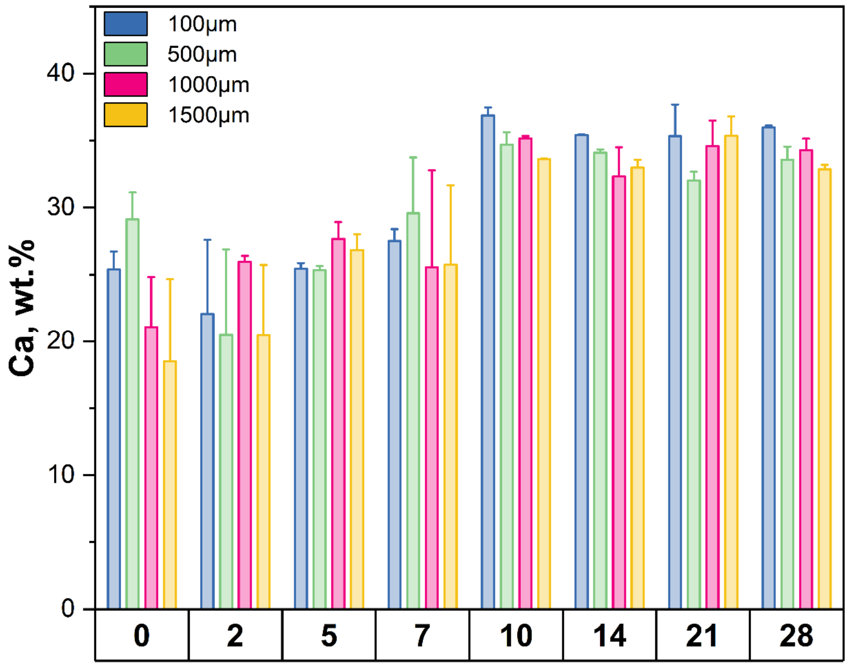

Figure S1. Ca amount dependence on the day of postnatal life and distance from the incisor's tip.

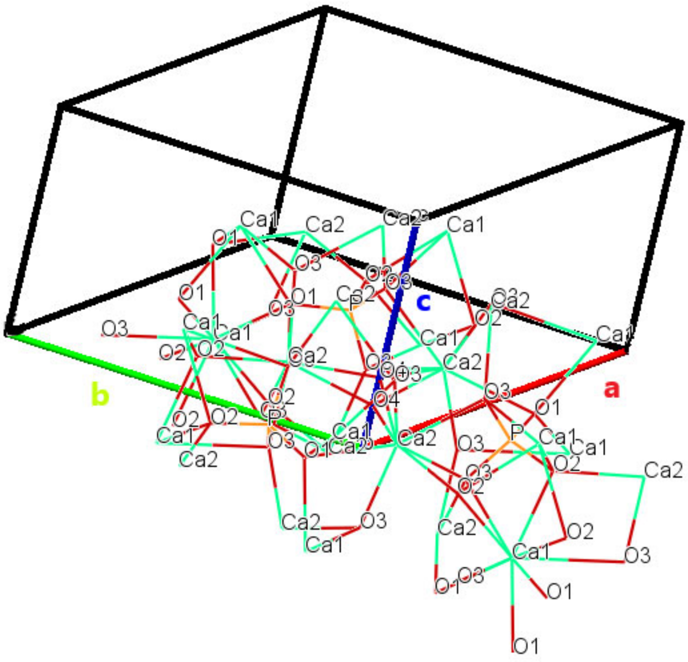

Figure S2. The chemical structure of synthetic hydroxyapatite with the selected cell and signed lattice parameters (Mercury 3.10.1); a – lattice parameter (length) in x direction, b – lattice parameter (length) in y direction, c – lattice parameter (length) in z direction

Table S3. Pearson's correlation coefficients between variables used in the MLP training.

Variable	dpn	Ca	P	Ca/P	C	Fe	FWHM	$a_{lattice}$	CI_{Raman}	CI_{FTIR}	$(C/P)_{FTIR}$	$(C/P)_{Raman}$	DMT_I	DMT_{II}
dpn	1,00													
Ca	0,65	1,00												
P	0,82	0,93	1,00											
Ca/P	-0,78	-0,32	-0,62	1,00										
C	-0,56	-0,31	-0,40	0,35	1,00									
Fe	0,83	0,71	0,75	-0,48	-0,63	1,00								
FWHM	-0,91	-0,72	-0,79	0,55	0,64	-0,89	1,00							
$a_{lattice}$	-0,88	-0,68	-0,74	0,52	0,62	-0,87	0,97	1,00						
CI_{Raman}	-0,40	-0,39	-0,40	0,28	0,35	-0,44	0,52	0,47	1,00					
CI_{FTIR}	0,57	0,46	0,46	-0,27	-0,48	0,60	-0,72	-0,63	-0,51	1,00				
$(C/P)_{FTIR}$	-0,90	-0,72	-0,80	0,58	0,64	-0,88	0,95	0,92	0,48	-0,68	1,00			
$(C/P)_{Raman}$	-0,75	-0,55	-0,61	0,48	0,55	-0,71	0,86	0,79	0,60	-0,74	0,78	1,00		
DMT_I	0,90	0,48	0,70	-0,80	-0,45	0,67	-0,73	-0,73	-0,28	0,36	-0,72	-0,59	1,00	
DMT_{II}	0,91	0,48	0,70	-0,79	-0,43	0,68	-0,73	-0,72	-0,25	0,35	-0,71	-0,57	0,98	1,00
